# Dural Venous Sinus Thrombosis Leading to Subarachnoid Hemorrhage

**DOI:** 10.7759/cureus.13497

**Published:** 2021-02-22

**Authors:** Harwindar Kumar, Sundas Ali, Jasvindar Kumar, Muhammad Nabeel Anwar, Romil Singh

**Affiliations:** 1 Neurology, Lady Reading Hospital, Peshawar, PAK; 2 Internal Medicine, Holy Family Hospital, Rawalpindi Medical University, Rawalpindi, PAK; 3 Internal Medicine, Khyber Teaching Hospital, Peshawar, PAK; 4 Medicine, Mayo Hospital, Lahore, PAK; 5 Critical Care, Mayo Clinic, Rochester, USA

**Keywords:** subarachnoid hemorrhage, dural venous sinus thrombosis, cerebral venous sinus thrombosis

## Abstract

Dural venous sinus thrombosis (DVST) leading to subarachnoid hemorrhage (SAH) is rarely reported in the literature. A 25-year-old primigravida with a history of pre-eclampsia presented with sudden onset headache, confusion, and loss of consciousness. Examination revealed bilateral equivocal planters and bilateral papillary edema. MRI and magnetic resonance venography (MRV) showed the right sinus thrombosis with elements of SAH. The coagulation profile was unremarkable. She was commenced on low molecular weight heparin with periodic monitoring of her Glasgow Coma Scale (GCS). Her condition started improving gradually. Repeat MRI and MRV after 10 days showed resolution of thrombosis and SAH. She was discharged with follow-up, and she was doing well on her recent visit two weeks later.

## Introduction

Dural venous sinus thrombosis (DVST) complicated by subarachnoid hemorrhage (SAH) is very challenging to diagnose [1]. DVST is seen in cumulative frequency in regular practice and generally presents with headache, nausea, vomiting, weakness, loss of vision, and seizure. DVST presenting with SAH signs and symptoms is not typical in the literature and has been documented as a potential complication of DVST [2]. Vascular lesion such as ruptured aneurysm is a common etiology of SAH; however, a small number of patients may have SAH due to DVST. Therefore, it is a matter of absolute importance to pick the signs of the suspected non-aneurysmal source of SAH, such as DVST. Herein we present a case of DVST leading to SAH in an adult female.

## Case presentation

**Figure 1:**
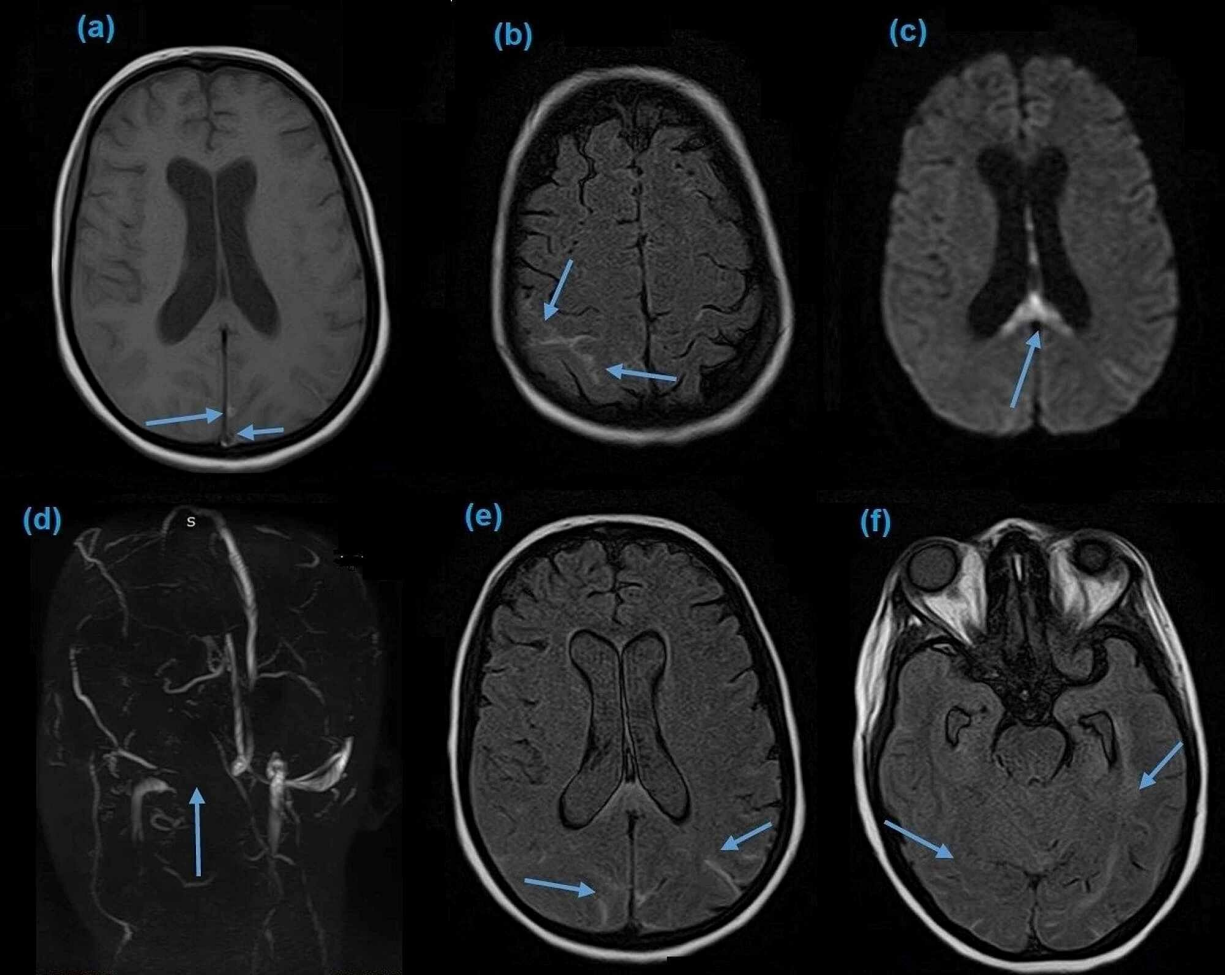
Axial images of the brain MRI showing DVST and SAH; high signal linear intensities in T1-weighted image, more prominent in the parietal lobe (a and b), diffusion restriction in the splenium of corpus callosum (c), loss of visualization of right sinus transverse on MRV (d), hyperintensity in bilateral sulci of the cortex (e), and hyperdense material filling the subarachnoid space (f). MRI: magnetic resonance imaging; DVST: dural venous sinus thrombosis, SAH: subarachnoid hemorrhage, MRV: magnetic resonance venography

A 25-year-old primigravida with a history of pre-eclampsia was brought to the emergency department with sudden onset of severe headache. The headache started two days after delivery. It was followed by confusion and loss of consciousness over a few hours. On initial evaluation, the patient was confused with a Glasgow Coma Scale (GCS) of 12/15, blood pressure of 130/90 mmHg, pulse rate of 82 beats/min, respiratory rate of 30/min, temperature 98°C, and oxygen saturation of 97 mmHg at room temperature. On physical examination, bilateral equivocal planters were noted with no other significant findings, and bilateral papillary edema on fundoscopy was seen. The rest of the review system was unremarkable. She underwent an urgent MRI with magnetic resonance venography (MRV) of the brain, which showed right transverse sinus thrombosis with an element of SAH (Figure 1).

**Figure 2:**
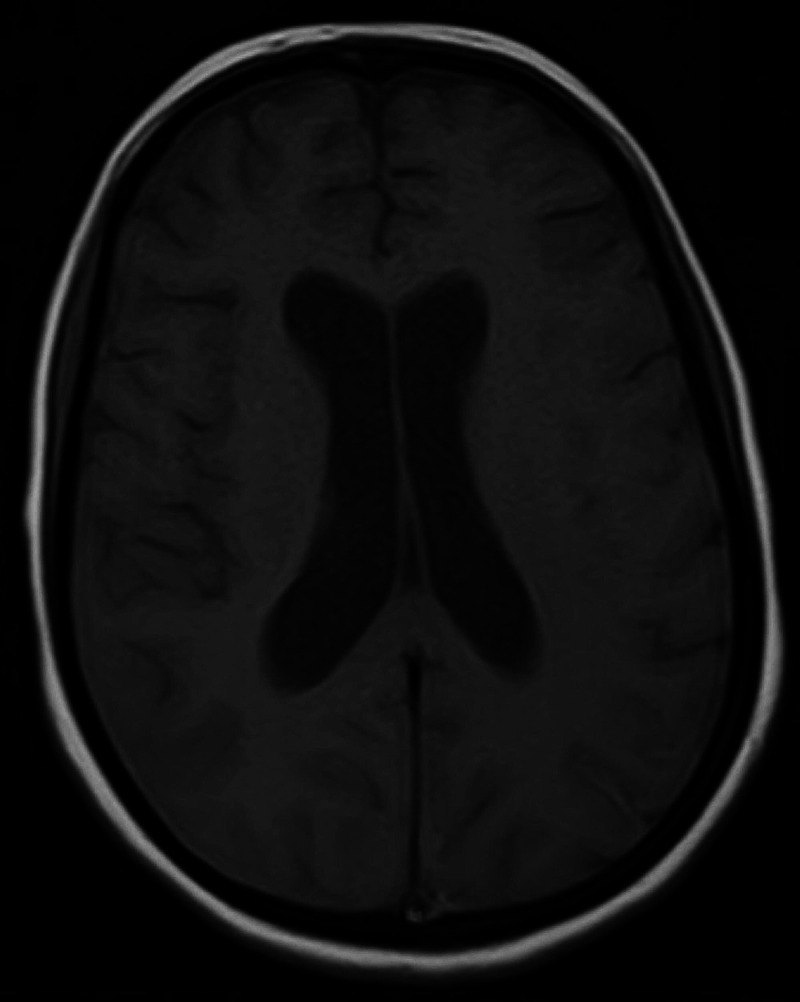
Axial image of the brain after 10 days of hospital admission showing resolution.

Coagulation profile, including thrombin time (TT), prothrombin time (PT), partial thromboplastin time (PTT), antithrombin III, fibrinogen, proteins C and S, antiphospholipid antibody titers, and homocysteine levels, were normal. She was started on a half dosage of low molecular weight heparin (LMWH) (60 mg subcutaneously once a day) with regular monitoring of her blood pressure and GCS. Her GCS started improving; therefore, she was continued on a half dose of LMWH for 10 days, and brain MRI was repeated after 10 days, which showed the gradual resolution of SAH (Figure 2). The quantity of LMWH was doubled, and warfarin was started after a full dose of LMWH. After improving her GCS and achievement of international normalized ratio (INR) of two to three, she was discharged with the advice of regular INR monitoring and follow-up to the outpatient department. On follow-up at six weeks, MRI with MRV was repeated, which showed thrombosis had resolved, although a mild filling defect was noted. She was kept on anticoagulants for one year, and on further follow-up at six months, she was doing well.

## Discussion

Dural venous thrombosis constitutes 1-2% of all strokes in adults, and DVST is an uncommon factor in causing SAH. In a study by Panda et al., about 4.4% of the patients with dural venous thrombosis were reported to have SAH [3]. Another study underlined that DVST was associated with 3% of SAH [4]. DVST has a broad spectrum of clinical manifestations ranges from headache with papilledema and neck stiffness to focal neurological deficit and seizures accompanied by 30-50% of affected patients, often followed by Todd’s paresis. DVST can be challenging to diagnose due to its wider clinical manifestations, and the presentation of a patient with acute SAH may further complicate the diagnosis. The superior sagittal sinus thrombosis may induce bleeding, leading to dilated vein rupture [2]. The area involved in hemorrhage varies from the arterial aneurysm. 

The exact mechanism of SAH is unidentifiable. It may involve the rupture of venous parenchymal hemorrhagic infarcts into the subarachnoid space. Another possibility is hypertension, as seen in our patient with eclampsia, which results in rupture of the dilated, thin-walled subarachnoid cortical veins lacking smooth muscle fiber [5]. DVST leading to a localized inflammatory response can also induce SAH, resulting in increased vascular permeability [5]. 

Diagnostic subtraction angiography (DSA) remains the diagnostic gold standard. The presence of acute SAH in the cerebral convexity warrants the dedicated vascular imaging of both dural venous sinuses and intracranial arteries. DSA remains standard for cerebral aneurysm detection in some cases, and it is still a part of systemic workup in SAH. The findings in DSA result in the diagnosis of DVST, even if it is previously unanticipated. Non-invasive angiographic techniques are far better in diagnosing the SAH caused by dural venous sinus thrombosis and replacing DSA. However, diagnosis of DVST is becoming more difficult, and even CT or MR angiogram focused on Willis' circle does not provide the perfect imaging of the venous system of distal arteries [6]. Hence, DVST is systematically measured in the SAH diagnostic workup of the cerebral convexity; it might be possible that DVST remains undiagnosed despite non-invasive angiographic procedures [7].

The management of venous SAH, which occurs secondary to DVST, varies considerably from arterial SAH. In patients with cerebral hemorrhage or intracranial hemorrhage, the usual systemic anticoagulation or localized thrombolysis, the first-line treatment for sinus thrombosis, is considered safe and effective, not deemed contraindication to heparin therapy. However, the presented case is SAH, not intraparenchymal hemorrhage. While heparin is long being safe for DVST treatment, its benefits were seen in a randomized controlled trial of 20 patients [8]. Besides, a second placebo randomized control study has suggested promising results using anticoagulants (low molecular heparin accompanied by oral anticoagulation) in which 60 patients were randomized to either LMWH followed by warfarin or placebo, but the differences were not statistically significant [9]. Venous sinus thrombosis presents up to a 48% mortality rate. Studies have estimated that with treatment, patients have up to a 62.5% full recovery rate. Heparin and Warfarin are used for systemic anticoagulation in the hopes of breaking up or reducing the clot. This is often used even when thrombolysis (surgical destruction of the clot) is anticipated [10]. Although no clear benefit is seen in a subsequent study, investigators continued to suggest non-significant trends in favor of anticoagulant therapy. In our case, we consider the initial low dosage of intravenous fractionated heparin, 60 mg once a day. Subsequently, on the 10th day, the dose was raised to 120 mg for a week after obtaining a CT scan supporting SAH deterioration. Oral warfarin was also added after no evidence of worsening of SAH. Our patient showed positive recovery in the effect of anticoagulation therapy.

## Conclusions

DVST should be considered in the differential diagnosis of patients presenting with acute SAH without the evidence of aneurysm, especially when the basal cistern is not involved. An important signal is the presence of blood in cerebral convexities in the absence of parenchymal lesions. This condition warrants immediate management and treatment with anticoagulation based on the stability of bleeding.
